# The cost of lower respiratory tract infections hospital admissions in the Canadian Arctic

**DOI:** 10.3402/ijch.v72i0.21595

**Published:** 2013-08-05

**Authors:** Anna Banerji, Val Panzov, Joan Robinson, Michael Young, Kaspar Ng, Muhammad Mamdani

**Affiliations:** 1 Li Ka Shing Knowledge Institute, St. Michael's Hospital, Toronto, ON, Canada; 2 Applied Health Research Centre (AHRC), Li Ka Shing Knowledge Institute, St. Michael's Hospital, Toronto, ON, Canada; 3 Stollery Children's Hospital, University of Alberta, Edmonton, AB, Canada; 4 IWK Health Centre, Dalhousie University, Halifax, NS, Canada; 5 Faculty of Medicine at the University of Toronto, University of Toronto, Toronto, ON, Canada

**Keywords:** Lower Respiratory tract infections (or LRTI), costs, Canadian Arctic, Inuit

## Abstract

**Background:**

Inuit infants who reside in the Nunavut (NU) regions of Arctic Canada have extremely high rates of lower respiratory tract infections (LRTIs) associated with significant health expenditures, but the costs in other regions of Arctic Canada have not been documented.

**Objective:**

This prospective surveillance compares, across most of Arctic Canada, the rates and costs associated with LRTI admissions in infants less than 1 year of age, and the days of hospitalization and costs adjusted per live birth.

**Design:**

This was a hospital-based surveillance of LRTI admissions of infants less than 1 year of age, residing in Northwest Territories (NT), the 3 regions of Nunavut (NU); [Kitikmeot (KT), Kivalliq (KQ) and Qikiqtani (QI)] and Nunavik (NK) from 1 January 2009 to 30 June 2010. Costs were obtained from the territorial or regional governments and hospitals, and included transportation, hospital stay, physician fees and accommodation costs. The rates of LRTI hospitalizations, days of hospitalization and associated costs were calculated per live birth in each of the 5 regions.

**Results:**

There were 513 LRTI admissions during the study period. For NT, KT, KQ, QI and NK, the rates of LRTI hospitalization per 1000 live births were 38, 389, 230, 202 and 445, respectively. The total days of LRTI admission per live birth were 0.25, 3.3, 2.6, 1.7 and 3 for the above regions. The average cost per live birth for LRTI admission for these regions was $1,412, $22,375, $14,608, $8,254 and $10,333. The total cost for LRTI was $1,498,232 in NT, $15,662,968 in NU and $3,874,881 in NK. Medical transportation contributed to a significant proportion of the costs.

**Conclusion:**

LRTI admission rates in NU and Nunavik are much higher than that in NT and remain among the highest rates globally. The costs of these admissions are exceptionally high due to the combination of very high rates of admission, very expensive medical evacuations and prolonged hospitalizations. Decreasing the rates of LRTI in this population could result in substantial health savings.

Inuit infants who reside in regions of Arctic Canada have extremely high rates of lower respiratory tract infections (LRTIs) (1–5) associated with significant health expenditures (6,7). The 4 regions in Canada where Inuit primarily live include the Northwest Territories (NT), especially along the Inuvialuit Settlement Region (ISR), Nunavut (NU) with its 3 regions Kitikmeot, Kivalliq and Qikiqtani (Baffin), Nunavik in northern Quebec and Nunatisavut in Labrador. Of these regions, most of the data pertaining to LRTI in the Canadian Arctic are from NU, specifically the Qikiqtani Region. Historically, this region has had LRTI admissions rates as high as 410 hospitalizations per 1,000 children <1 year in 1974 (5), with more recent rates of 315 per 1,000 live births in the first year of life (7). In a prospective surveillance of LRTI in infants less than 6 months of age in the Qikiqtani Region, LRTI rates were as high as 484/1,000 (3). A retrospective study of infants admitted from NT and Kitikmeot over a 5-year period documented LRTI hospitalization rates of admission of 590 per 1,000 live births in the Kitikmeot Region. This region also had higher rates of infants requiring admission to intensive care units (ICU) compared to NT (4). Studies in NU have documented that the rates of admission to the ICU range from 10% in the Kitikmeot Region (4) to 12% in the Qikiqtani Region in the first year of life (2,3). In a retrospective analysis, the rate of LRTI admissions in Nunavik was 305 per 1,000 child-years.

As there is only one regional hospital in NU, LRTI admissions in NU usually occur outside the territory, involving medical air evacuation, an expensive mode of transportation over long distances. LRTIs including medical air evacuations account for significant health expenditure in NU and are among the most significant health issues facing this population (6,7). One study in the Qikiqtani Region calculated the costs relating to LRTI as $14,273 per admission to the regional hospital and $45,688 to the tertiary hospital (6). In other regions of Arctic Canada, there has been no comparison of LRTI admissions rates and associated costs. The objective of this study was to estimate and compare the rates and costs of LRTI admissions in NT and the 3 regions of NU and Nunavik through a hospital-based surveillance of infants less than 1 year of age over a period of 18 months.

## Methods

The Canadian Arctic has 4 regions that are heavily populated by Inuit comprising: (a) ISR in the NT, (b) NU with 3 regions of Kitikmeot, Kivalliq and Qikiqtani, (c) Nunavik in northern Quebec, and (d) Nunatsiavut in Labrador. Approximately 80–90% of the population in NU and Nunavik are Inuit, while in NT Inuit represents 10% of the population and primarily resides in the ISR regions. The rest of the NT population comprise 31% First Nations, 9% Métis and 50% non-Aboriginal (8).

**Fig. 1:**
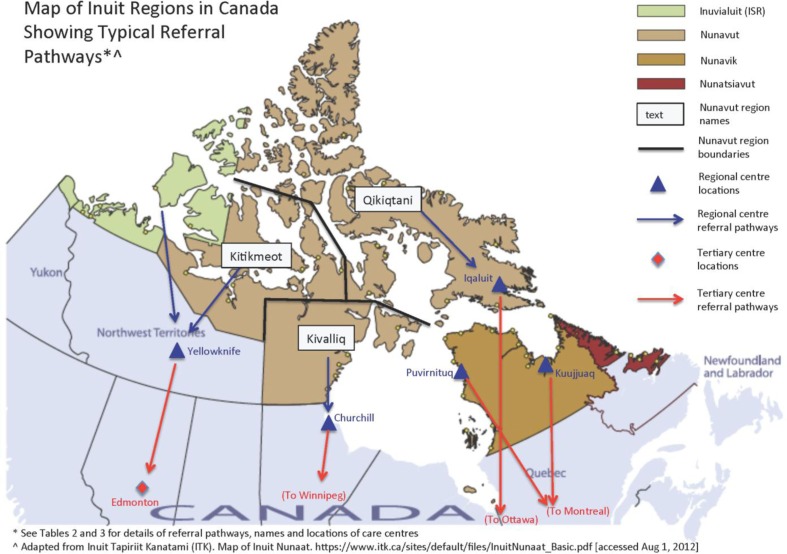
Map of Inuit regions in Canada and typical referral patterns in 2009.

This was a hospital-based surveillance with the following inclusion criteria: LRTI admission for an infant less than 1 year of age, residing in NT, NU or Nunavik and admitted from 1 January 2009, for 1 year. Funding was extended for an additional 6 months up to 30 June 2010. We compared rates of LRTI admissions and costs across 5 regions: NT, Kitikmeot, Kivalliq and Qikiqtani Regions of NU and Nunavik. In addition, we included data on the ISR region of NT because of its high proportion of the population that are Inuit. All of the major regional and tertiary centres that served the populations participated in this study (Fig. 1). Nunatsiavut declined to participate in the surveillance study citing low birth numbers and perceived low rates of LRTI.

The surveillance included 5 regional hospitals: Stanton Regional Hospital (SRH) in Yellowknife, NT; Churchill Health Centre (CRH) in Churchill Manitoba; Inuulitsivik Health Centre (IHC) in Puvirnituq, Quebec (QC); Ungava Tulattavik Health Centre (Kuujjuak, QC); and 4 tertiary hospitals: Stollery Children's Hospital in Edmonton, Alberta’ Children's Hospital of Winnipeg in Winnipeg, Manitoba; Children's Hospital of Eastern Ontario (CHEO) Ottawa, Ontario; and Montreal Children's Hospital, Montreal, QC.

Each hospital had a site investigator and a research nurse who prospectively identified infants who met the inclusion criteria and obtained informed consent and implemented a questionnaire to the parent or guardian of the infant in all regions except Qikiqtani. Unanticipated delays in obtaining administrative approval in the Qikiqtani Region necessitated data collection through a retrospective chart review for infants from that region. In addition, all admission records for infants who were less than 1 year of age at each site were reviewed at the end of the study to identify LRTI cases that were missed by the surveillance. This study enrolled infants of all ethnicities who resided in Arctic regions.

### Costs

Canada has universal health care coverage, which is generally not-for-profit. In general, the federal government through the Non-Insured Health Benefit (NIHB) program covers the cost of medical transportation for the Inuit, while the Provincial or Territorial governments cover the direct medical costs. Most Inuit infants live in rural communities where the nursing station is the sole medical facility. The perspective of the cost analysis was that of the public payer and the time horizon spanned the duration of our study. Given the short time horizon of our study, costs were not discounted. All costs are reported in 2011 American dollars (1 US=1 Canadian). The total cost of LRTI admissions included transportation, hospital stay, physician fees and accommodation costs, which were obtained directly from the territorial or regional governments and hospitals. An infant who was transferred to another hospital was counted as one admission. Outpatient follow-up costs were not included in the analyses. In addition, the cost of medications or complications such as high-frequency oscillation ventilation (HFOV), items such as chest tubes for surgical procedures and the cost of readmission outside the study were not included. The cost of discharged infants who remained in boarding homes for convalescence or who were awaiting transportation was also not included.

### Transportation costs

Transportation costs included the cost of medical evacuations (medevac) from the residential community to regional and/or tertiary hospitals and return travel back to their residential community on a scheduled flight. The cost of the medevac flight and personnel was obtained directly from the contracted companies. Cost of return travel back to the residential community was based on the cost of an economy-scheduled flight for 1 parent or guardian and was obtained from the commercial airlines.

### Hospital costs

Hospital stay costs were calculated based on the length of stay and estimated hospital per diem rates differentiated by ward. ICU stay costs were obtained from the hospital's finance department or from the regional governments. Days in hospital (length of stay) were all considered to be due to LRTI, recognizing that a small number of stays could be prolonged due to unrelated diagnoses. The Emergency Room (ER) assessment fee, which included hospital and physician fees and all procedures, was estimated at $700 for a tertiary centre and $1,000 for a regional centre. The ER assessment fee was calculated only once per admission, even if they were subsequently transferred to a different institution.

Physician fees were estimated from the Ontario Schedule of Benefits for Physician Services or Ontario Health Insurance Plan (OHIP) fees, which was mid-range for the regions, (9) and did not include any after-hours or emergency call-in fees. The initial assessment included consultation fees (paediatric rates) for admission to the hospital ward followed by daily visit fees. A 30% premium was added to physician fees incurred at a regional hospital to reflect a northern allowance. If an infant was admitted to an ICU, physician ICU daily fees were included which varied by the length of stay and if airway management was required (9).

#### Accommodation costs

Accommodation at a hotel/hostel for 1 parent or guardian while the subject was admitted to a tertiary centre was estimated as $150 per day.

The study obtained ethics and internal review from all hospitals and the Applied Health Research Centre (AHRC) at St. Michael's Hospital. The study was licensed though the Aurora Research Institute in the NT and the Nunavut Research Institute. In addition, before commencing the study, there was extensive consultation with Inuit advocacy groups.

## Data analysis

All data were initially entered into an Excel 2010 spreadsheet and then imported into SAS for analysis. The estimated birth cohort in this 18-month period was obtained from vital statistics registry for births 2009 and half of 2010 (10). Admissions and length of stay were compared for NT, Kitikmeot, Kivalliq and Qikiqtani and Nunavik; however, data were also recorded for ISR and all NU. Each admission to a regional or tertiary hospital or to both (in the case of a transfer) was considered 1 admission, with readmissions were counted separately.

The number of LRTI admissions per 1,000 live births and the days of hospitalization per 1,000 live births were determined.

For each region, the median and range of the length of hospitalization stay was reported. In addition, the length of stay (total hospitalization days) was calculated for regional and tertiary hospitals and combined for LRTI admissions over the study period. The number of admissions who were hospitalized for more than 3 weeks was also documented.

The total hospitalization days were compared for regional, tertiary and combined hospitalizations in each region using the Kruskal-Wallis test, which noted that there was a difference between the total hospitalization days in each region, where NT was lower than the other regions. Poisson regression was used to compare the rates of LRTI and the total days of hospitalization per live birth.

For each of the regions, the average cost per admission was calculated for all costs and subdivided into the costs for average transportation costs (medevac only and total including return) and for inpatient costs. The total costs were divided into subcategories: transportation, inpatient, physician fees, and the ER and accommodation fees were combined. To estimate the cost of LRTI per live birth, the total costs were divided into the estimated live births over the study period.

## Results

There were 513 LRTI admissions (409 to regional centres alone, 69 to tertiary centres alone and 35 to both) by 438 unique infants during the study period. Table I documents LRTI admissions across the regions. Rates of admission in NT (38 per 1,000 live births) were considerably lower than the regions of NU or Nunavik (Table I) (p<0.0001 and were highest in Nunavik at 445 per 1,000 live births). The median length of stay was comparable among the groups; however, NU and Nunavik had several infants with prolonged hospitalization (up to 216 days). It is of note that the median length of stay in this population ranged from 5 to 6 days overall but from 6 to 21 days for those who were admitted to tertiary centres. Up to 13.5% of the admissions from the Kivalliq Region were hospitalized for more than 3 weeks.

**Table I:** Length of stay and LRTI hospitalization rates by region for infants <1 year of age residing in the Canadian Arctic from 1 January 2009 to 30 June 2010

Territory	Northwest Territories (NT)		NUNAVUT				Nunavik
			
Region	NT (all)	ISR only	Nunavut (all)	Kitikmeot	Kivalliq	Qikiqtani	Nunavik
Estimated births from 1 January 2009 to 30 June 2010*	1,061	168	1,291	180	388	723	375
LRTI hospitalization rate per 1,000 live births-year	38	18	236	389	230	202	445
**Total LRTI hospitalizations**							
Length-of-stay median (range)	5 (2–57)	6 (5–8)	6 (1–216)	7 (1–36)	6 (1–216)	6 (2–156)	5 (1–96)
Total hospitalization (days)	270	19	2,882	598	1,022	1,262	1,132
**Regional LRTI hospitalizations**							
Number of admissions	41	3	258	67	57	134	148
Length-of-stay median (range)	4 (1–10)	6 (5–8)	5 (1–42)	7 (1–22)	5 (1–42)	5 (1–32)	4 (1–16)
Total hospitalization (days)	156	19	1,618	487	381	750	686
**Tertiary LRTI hospitalizations**							
Number of admissions	6	0	71	5	40	26	28
Length-of-stay median (range)	11 (2–56)	0	6 (2–216)	21 (14–36)	6 (2–216)	11.5 (3–153)	9 (2–96)
Total hospitalization (days)	114	0	1,264	111	641	512	446
Total hospitalization (days) per live birth	0.25	0.1	2.2	3.3	2.6	1.7	3.0

* From Statistics Canada the birth denominator is: births in 2009 plus half births in 2010. Where data was not available, we referred to the Ministry of Health for each region.

Among the 3 regions, the LRTI hospital days per live birth in NT (0.25) were significantly lower that for Nunavik (3) and Kitikmeot (3.3) (p<0.0001). Total cost per LRTI admission varied widely; from $23,203 in Nunavik to $63,686 in the Kivalliq Region, where medical evacuation costs accounted for much of this variability between regions (Table II). The majority of costs were related to inpatient hospital and ICU costs in all regions except ISR. Transportation ranged from 17.9% of total costs in the Kivalliq Region to 55% in the ISR. Medical evacuations accounted for the majority of transportation costs, which were as high as $38,500 one way. Physician and other fees (emergency and accommodation fees) were minor contributors to the total costs. NT had considerably lower costs for LRTI admission per live birth compared to the regions of NU and Nunavik, which ranged from $1,412 in NT to $22,375 in Kitikmeot Region.

**Table II:** Total LRTI admissions, along with average and total costs for LRTI hospitalizations in infants less than 1 year age admitted between 1 January 2009 and 30 June 2010 in the Canadian Arctic

Territory	Northwest Territories (NT)		Nunavut				Nunavik
			
Region	NT (all)	ISR only	Nunavut (all)	Kitikmeot	Kivalliq	Qikiqtani	Nunavik
LRTI admissions (n)	41	3	305	70	89	146	167
**Average cost per LRTI hospitalization**							
Medical evacuation, average (range)	$7,865 (0–$35,517)	$20,859 ($18,924–$23,273)	$12,086 ($0–38,494)	$19,378 ($14,000–$38,494)	$10,151 ($2,776–$20,846)	$9,770 (0–$34,441)	$5,681 (0–$25,18)
All transportation average (range)	$8,285 ($0–36,913)	$22,000 ($19–924–24,564)	$13,392 ($0–41,175)	$21,086 ($15,211–41,175)	$11,377 ($3,448–2,250)	$10,392 (0–$36,902)	$6,121 (0–$26,667)
Inpatient costs Average (range)	$25,7,839 ($4,680–$344,108)	$14,820 ($11,700–$187,20)	$27,753 ($1,253–$1,130, 112)	$34,097 ($3,510–$219,708)	$48,851 ($2,609–$1,130, 112)	$27,281 ($2,132–$808,323)	$14,724 ($1,253–$338,688)
All costs	$36,542	$38,629	$55,265	$57,535	$63,686	$40,873	$23,203
**Total costs for all LRTI hospitalizations**							
Total transportation cost (%)	$339,674 (22.7)	$65,967 (54.9)	$4,084,596 (26.1)	$1,476,019 (36.6)	$1,012,527 (17.9)	$1,596,050 (26.7)	$1,022,264 (26.4)
Total inpatient costs (%)	$1,060,782 (70.8)	$44,460 (37.0)	$10,717,638 (68.4)	$2,386,803 (59.3)	$4,347,741 (76.7)	$3,983,094 (66.7)	$2,458,896 (63.5)
Physician fees (%)	$81,576 (5.4)	$5,431 (4.5)	$681,784 (4.4)	$148,741 (3.7)	$217,638 (3.8)	$315,405 (5.3)	$331,020 (8.5)
ER and accommodation (%)	$16,200 (1.1)	$5,431 (3.6)	$178,951 (1.1)	$15,900 (0.4)	$90,151 (1.6)	$72,900 (1.8)	$62,701 (1.6)
All costs	$1,498,232	$120,189	$15,662,968	$4,027,463	$5,668,056	$5,967,449	$3,874,881
Average cost per live birth	$1,412	$690	$12,132	$22,375	$14,608	$8,254	$10,333

## Discussion

LRTI hospitalizations are a significant health issue for Canadian infants who reside in the Arctic with substantial cost implications. Furthermore, there are very significant regional differences in both hospitalization rates and the cost of managing an LRTI. For example, the rates and cost of LRTI admission for NT were about one tenth of those in NU and Nunavik. While the majority of the population in NU and Nunavik is Inuit, Inuit represent only 10% of the population in NT which raised the question of whether genetics may have a contributing role in this region in addition to the environmental factors, such as decreased access to health care, overcrowding, lack of breastfeeding, exposure to cigarette smoke, and other factors such as poverty (1). A case control study in the Qikiqtani Region found that an Inuit infant with 4 Inuit grandparents had a nearly 4-fold higher risk of LRTI admission compared to mixed and non-Inuit children (1). A retrospective chart review comparing rates of LRTI in Inuit communities in NU versus primarily First Nations infants in NT demonstrated a large discrepancy, despite similar geographic, social and economic conditions (8). However, in this study, the ISR region also had lower rates, which were more consistent with NT than with other regions even though the sample size was small.

The health care costs associated with LRTI hospitalizations in infants in NU and Nunavik are extremely high. In Nunavik, an estimated 45% of the birth cohort were admitted to the hospital with an LRTI which is the highest proportion described in the literature. However, as the distances travelled for medical evacuations were relatively low compared to NU, the cost of LRTI hospitalizations per live birth was less than in NU. In NU, most admissions for LRTI were admitted outside the territory, often at a great distance and at great expense. In fact, half of the federal NIHB contribution to health care in NU from 1996 to 2006 went on medical transportation (11). The fact that each infant born in NU has an average or 2.2 days of LRTI hospitalization at a cost of $12,000, and 3 days at a cost of $10,000 in Nunavik, respectively, should be a call for action for strategies to reduce LRTIs in these regions.

It appears that infants from the Arctic have prolonged hospitalization, especially in the Kivalliq Region where 13.5% were hospitalized for more than 3 weeks compared to a large Canadian study with a median of 3.1 days in 2000 (12). Many of the infants in this study were admitted to ICU and often required prolonged periods of mechanical ventilation (including HFOV). The costs of complications from prolonged hospitalization with possible sequelae are not captured in this study (13,14). The study also does not account for the cost or impact of parental stress, family separation and loss of employment.

Respiratory syncytial virus (RSV) is the most common virus associated with LRTI admissions globally, where it is estimated that RSV is responsible for 64 million cases and 160,000 deaths each year (15). Although there are no current economical evaluations for the cost of LRTI admissions in Canada, an economic evaluation of RSV in Canada for children less than 4 years of age found that the RSV cost was almost $18 million in 1994. The largest component of this cost was inpatient medical care (62%), while ambulatory costs were 38% (12). In contrast, this study conservatively estimates costs for NU for LRTI hospitalizations of patients less than 1 year of age at $15.6 million [45% was related to RSV (Banerji, unpublished)]. The contrast is even greater when considering the annual number of births in Canada is approximately 380,000, while in NU it is just above 800 or approximately 0.2% of the Canadian birth rate (10). In addition, studies in Canada have documented a rate of outpatient LRTI visits (12,16) and conclude that the costs related to outpatient LRTI management can be substantial.

Optimal measures to decrease the incidence of LRTI admissions in this population remain unstudied. Our previous study demonstrated that the use of palivizumab in term infants is potentially cost effective in communities with consistently high rates of RSV admissions. In the Qikiqtani Region, RSV rates as high as 328 per 1,000 live births occur in the RSV season for infants less than 6 months of age in communities without a hospital (7). As the rates and costs associated with RSV were demonstrated to be exceedingly high, prevention of RSV with monoclonal antibody (RSV) in a budget impact analysis demonstrated that universal prophylaxis of infants less than6 months of age in these rural communities would actually result in cost savings. Other measures that may decrease rates of LRTI include decreasing exposure to cigarette smoke, enhancing breastfeeding, addressing overcrowding (1), increasing influenza immunization of infants over 6 months of age and their household contacts.

## Limitations

For several reasons, we believe that the costs in this study are conservative inpatient estimates. There were no additional costs added for evening or weekend assessment fees, emergency visits and resuscitations. In addition, we did not include the cost of surgery, boarding accommodation near the regional hospital during convalescence or drugs. A significant number of infants required very expensive interventions such as HFOV. LRTI charts were reviewed in all hospitals, but we are aware that milder cases of LRTI requiring short stay may have occurred in Inuvik NT. In the spring of 2010, a new health centre opened in Rankin Inlet, Kivalliq, which re-routed infants away from Churchill. In addition, we are aware of 3 admissions to tertiary centres ICU outside the study, when the usual tertiary centre ICU exceeded capacity. We estimate that we captured approximately 90% of the LRTI admissions during this time period. This study was over an 18-month period, which would normally capture 2 RSV seasons, overestimating the costs of LRTI. Unexpectedly, the rates of LRTI admission in the first year were almost identical to the rates over 18 months and the 2010 RSV season was delayed outside the study.

## Conclusion

LRTI admission rates in NU and Nunavik are much higher than in NT and remain among the highest rates globally. The costs of these admissions are exceptionally high due to the combination of very high rates of admission, very expensive medical evacuations and prolonged hospitalizations. Decreasing the rates of LRTI in this population could result in substantial health savings that could be redirected to prevention of other health issues. Prevention strategies to reduce the acute and long-term consequences of LRTI in Canadian infants residing in these regions are needed.
